# Opportunity-Reduction Supervision Strategies With Domestic and Family Violence Probationers and Parolees

**DOI:** 10.3389/fpsyg.2022.878544

**Published:** 2022-06-02

**Authors:** Lacey Schaefer, Gemma C. Williams, Emily Moir

**Affiliations:** ^1^School of Criminology and Criminal Justice, Griffith Criminology Institute, Griffith University, Mount Gravatt, QLD, Australia; ^2^School of Law and Society, University of the Sunshine Coast, Maroochydore, QLD, Australia

**Keywords:** domestic and family violence, probation and parole, community corrections, crime opportunities, opportunity reduction, environmental criminology, Environmental Corrections

## Abstract

Many forms of supervision strategies traditionally utilized by probation and parole officers emphasize service brokerage, case management, and compliance. Conversely, there is a growing evidence-base that demonstrates how community corrections practices can be (and have been) improved through supervision frameworks of behavior change oriented around criminogenic needs. Toward this end, recent advances in penology have applied the tenets of environmental criminology theories to community corrections practices, seeking to identify and modify each individual’s opportunity-based risks for reoffending. In this article, using data from an Australian experimental trial, we explore the utility of an “Environmental Corrections” approach to the supervision of domestic and family violence perpetrators serving probation and parole orders, an offending cohort with growing political and public pressures. Quantitative analyzes indicate that this opportunity-reduction supervision framework was effective in reducing recidivism among all offenders. Amongst probationers and parolees on community corrections orders for domestic and family violence offenses only, rates of reoffending were 15.41% lower for offenders at the treatment site compared to the control site, although this difference was not statistically significant. A thematic analysis of semi-structured interviews with these clients highlights that through the Environmental Corrections trial, they learned strategies for identifying, avoiding, and resisting opportunities to reoffend. Combined, this evidence suggests that opportunity-reduction supervision tactics may hold promise for limiting recidivism amongst domestic and family violence perpetrators, although further research is required.

## Introduction

Given the substantial number of people affected by the corrections system, we must question whether agencies, communities, and clients are benefiting from this arrangement as expected (Cullen et al., 2017). The massive growth in the world’s prison populations has had enormous consequences for community corrections; more people are placed on community-based orders as a diversion from incarceration and more people are supervised on parole following their discharge from custody (Phelps, 2013; Schaefer and Brewer, 2022). Given the growth in the number of individuals under correctional control, reoffending is a significant concern for the criminal justice system and communities alike. For instance, 62% of released prisoners in the US were arrested within 3 years of release (Durose and Antenangeli, 2021), 29% of released prisoners in England and Wales were reconvicted within 1 year (Ministry of Justice, 2020), and in Australia roughly half of released prisoners return to custody within 2 years (Australian Government Productivity Commission, 2021). Rates of reoffending have remained a stubborn penological problem in many jurisdictions around the world, despite substantial growth in the evidence-base of how to effectively supervise and rehabilitate correctional clients (Cullen et al., 2017).

Parallel to this problem has been the heightened political and policy attention paid to domestic and family violence (DFV), facilitated by movements such as ‘‘Me, too’’ and ‘‘March 4 Justice’’ (social justice initiatives that aim to highlight the unacceptably high rates of sexual, physical, emotional, and financial abuse against females).^1^ Official figures detail the startling prevalence of these offenses. Estimates by the World Health Organization [WHO] (2021), for example, indicate that globally, roughly one in three women (30%) have been the victim of physical or sexual violence at least once in their life since the age of 15. These figures are mirrored in Australia, where two in five adults (39%) reported an incident of physical or sexual violence since the age of 15 (Australian Bureau of Statistics, 2017); one in six women reported experiencing physical or sexual violence by a cohabitating partner since the age of 15 (Australian Institute of Health and Welfare, 2018), and there is evidence that rates of male DFV victimization are increasing (Gleeson, 2020). Although experts acknowledge that the true extent of DFV is unknown (Mouzos and Makkai, 2004) and violence can be bidirectional, data reveal that these offenses are highly gendered, disproportionately affecting women (along with children and other vulnerable groups, such as the elderly and disabled; Australian Institute of Health and Welfare, 2019).

Combined, these social problems produce enormous implications for victims and families but also for public health and community safety more broadly. For instance, a study in New South Wales identified that roughly one-fifth (19.7%) of final Apprehended Domestic Violence Orders (ADVOs, which are court orders made to protect threatened individuals from violence or threats of harassment from a spouse, *de facto* partner, ex-partner, family member, carer, or a member of the household) were breached (Poynton et al., 2016). Further investigation of the characteristics of ADVO breaches reveals that a significant portion of these individuals receives community supervision (15.7%) or custodial correctional (12.4%) penalties (Trimboli, 2015). Figures further highlight the recidivistic and dangerous nature of these offenses; of the persons who appeared in New South Wales courts in 2013 that involved a proven ADVO offense, more than half (53.3%) had at least one prior proven violence offense (namely assault and stalking), and more than one-quarter (28.7%) had previously breached an ADVO (Trimboli, 2015). In the Australian state of Queensland, more than 30,000 DVOs were breached in 2019, an increase from around 19,000 breaches just 5 years prior (Cartwright, 2021), and almost five times the number since 2001 (Lynch, 2020).^2^ These issues have likely been exacerbated by COVID-19, with victim surveys revealing that the pandemic has coincided with the onset or escalation of DFV (Boxall et al., 2020; Gleeson, 2020), and practitioners and policymakers referring to the increased violence against women as a “shadow pandemic” (Carrington et al., 2021).

Accordingly, responding to DFV has become a national priority in Australia, evidenced in legislative and policy frameworks such as the *National Plan to Reduce Violence against Women and their Children 2010–2022* (Council of Australian Governments, 2011).^3^ One of the realities of this landscape that government sectors must grapple with is how to manage these offenses. Apart from prevention and public education strategies, the criminal justice system is tasked with apprehending perpetrators and allocating and administering punishments, with corrections agencies responsible for the custodial and community supervision of clients who have DFV as their index offense or have a related order [e.g., a probationer serving a community sentence for drugs simultaneously having a Domestic Violence Order (DVO) against them]. The public scrutiny and political pressures of managing DFV offenders have likely created additional strains on an already overwhelmed system. As such, we must question the effectiveness of our approaches to offender management (Cullen et al., 2017; Schaefer and Brewer, 2022), including specific investigations of how best to meet the challenges presented by DFV perpetrators under community supervision (Crowe et al., 2009; Spencer et al., 2020). In this article, we pursue this objective. Using data from an experimental trial of a new model of probation and parole, we utilize a mixed-methods approach to explore whether an opportunity-reduction supervision framework has utility for DFV perpetrators serving community corrections orders.

## Literature Review

Although there are effective community corrections practices and an amalgamation of empirical support is building evidence-based practices in probation and parole (e.g., MacKenzie, 2006; Bonta et al., 2008; Andrews and Bonta, 2010; Trotter, 2013; Chadwick et al., 2015), these strategies and activities are not routinely utilized and widespread success is rarely observed (Schaefer et al., 2016; Schaefer and Brewer, 2022). As described by Cullen, Jonson, and Mears, their recommendation that community corrections be reinvented entirely around an evidence-base is “pregnant with the dismal truth that most supervision is guided by something else…[such as] practices rooted in misguided personal insights and bureaucratic convenience” (2017, p. 55). This apparent failure to use the empirical evidence of “what works” to guide offender supervision may be due to any number of issues (e.g., poor program fidelity, staff burnout and stress, no usable tools for converting evidence to practices, the absence of a guiding framework for orienting the actions of frontline staff; Latessa et al., 2002; Gleicher et al., 2013; Schaefer et al., 2016; Schaefer and Williamson, 2017; Schaefer and Brewer, 2022). Whatever the reason, the current state of probation and parole practices produces logical questions about whether an ideological transformation in community corrections is required.

There is a growing evidence-base of effective practices in probation and parole, with individual and meta-analytic studies highlighting useful supervision- and intervention-focused applications that have helped to improve processes and outcomes alike (MacKenzie, 2006; Andrews and Bonta, 2010; Chadwick et al., 2015). These advances notwithstanding, some scholars have argued that from the “nothing works” era of the 1970s to the present, “there has been a theoretical crisis in corrections,” whereby practices are a theoretical or logically mis-specified due to criminologists and policymakers who have “lost faith in rehabilitation but never gained faith in punishment” (Cullen and Jonson, 2016, p. 36). The lack of an ideological consensus for organizing frontline practices has produced aimless and generic “case management” frameworks that emphasize efficiency above effectiveness (Bull, 2010; Day et al., 2012), which has been exacerbated by rising caseloads and dwindling resources (Cullen and Jonson, 2016; Schaefer et al., 2016). Again, although some agencies routinely implement the “what works” aspects of community corrections, such efforts are rarely systematic or widespread (Cullen et al., 2017; Schaefer and Brewer, 2022). A review of Queensland’s parole system, for instance, described community corrections as “antiquated and emaciated,” requiring significant reforms “for the protection of the community” (Queensland Parole System Review, 2017, p. 1). Such criticisms are not new. More than 2 decades ago, the Reinventing Probation Council suggested that “agencies must start thinking outside the box for public safety, and design supervision strategies and programs for crime prevention and community betterment” (2000, p. 19).

Toward this end, Cullen et al. (2002) proposed that community corrections practices could be fundamentally reoriented by focusing on an oft-neglected element of the recipe for crime: opportunity. They suggest that environmental criminology may provide the theoretical framework for directing the goals and means of community corrections. Prior to describing an empirical investigation of the utility of such an approach to the supervision of DFV probationers and parolees, in the subsections that follow, we first identify the philosophies and practices embodied by “traditional” models of community corrections, then outline recent shifts toward opportunity-reduction frameworks.

### Traditional Models of Supervision

Traditionally, probation and parole supervision has been oriented around two philosophies—the control of propensity and deterrence tactics—both of which exhibit conceptual shortcomings that impact their capacity for limiting reoffending (Whetzel et al., 2011; Miller, 2014; Schaefer et al., 2016). First, authorities frequently aim to *control* (rather than change) individuals’ propensity for further offending. For instance, offenders are routinely directed to abstain from alcohol, even when alcohol may not be related to an individual’s risk profile or when their offending and substance use are caused by some spurious third factor (such as deficient impulse control). Even amongst the initiatives that are described as “treatment” in community corrections, unfortunately, these efforts are often centered around non-criminogenic needs (i.e., the intervention is focused on things that do not actually cause that person’s offending; Latessa et al., 2002) or are better described as service brokerage (e.g., putting clients in touch with accommodation providers; White and Graham, 2010; Hanser, 2013; Schaefer and Brewer, 2022). Although a noble goal on the surface, these efforts are not meaningfully rehabilitative, with little of the supervision process itself being oriented around more substantive correctional intervention (Solomon et al., 2005; Taxman, 2011; Smith et al., 2012; Raynor and Vanstone, 2015).

Second, probation and parole orders are often characterized by ineffectual threats of punishment for non-compliance with generic behavioral restrictions or prescriptions rather than the unique reoffending risks to which each supervisee is vulnerable (MacKenzie, 2006; Cullen and Jonson, 2016). Rules are poorly defined, monitored, and enforced, thereby hampering any possible deterrent effects we may hope to observe (Cullen et al., 2002; Schaefer et al., 2016). Meetings are organized around conversations that focus on the offender’s compliance with these vague conditions; not only does this distract officers from activities that could facilitate behavioral change (Bonta et al., 2008; Taxman, 2008; Bourgon et al., 2011; Lovins et al., 2018; Schaefer and Brewer, 2022), it further emphasizes the control of conditions that may be only loosely tied to reoffending risk (Schaefer et al., 2016), with evaluations showing “little evidence that deterrence-oriented community corrections reduces recidivism on a reliable basis” (Cullen et al., 2017, p. 29).

Combined, these misguided and poorly operationalized philosophies result in contemporary probation and parole practices that are focused on case management instead of crime prevention (Bonta et al., 2008; Pew Center on the States, 2008; Burrell, 2012). As noted by Cullen and Gilbert, both historically and contemporarily, “correctional officials get paid to maintain order and not to rehabilitate” (2013, p. 211). The prioritization of order compliance and completion is thus inherently limited in the ability of community correctional supervision to effectively discourage recidivism (Cullen, 2002; Taxman, 2008, 2011; Schaefer and Brewer, 2022). It is an unfair conclusion to assert that “nothing works” in the routine administration of community corrections, as there is a growing body of evidence about how recidivism can be reduced amongst probationers and parolees; it may rather be the case that agencies have failed to (properly or widely) implement the “what works” evidence into everyday practices (Cullen et al., 2017).

### Opportunity-Reduction Supervision

Stemming in part from a recognition of these limitations to routine probation and parole practices, scholars have called for community corrections to “be based on effective criminological research and theory” (Cullen et al., 2002, p. 30; see also Latessa et al., 2002; Cullen et al., 2017). Toward this end, Cullen et al. (2002) conceived that the theoretical frameworks, evidence of effectiveness, and practical utility of environmental criminology (and crime science more broadly) may provide an advance for the philosophies and practices used in probation and parole.

#### Environmental Criminology

Environmental criminological theories differ from other explanations for criminal behavior in that they reframe the problem: Rather than exploring the roots of criminality, environmental criminology questions the conditions that make a crime occur (Clarke, 2010). The criminological truism that crime clusters in time and space has drawn scholars’ attention to the features of those places that attract, generate, and facilitate offending (Schaefer, 2021). Although environmental criminology is comprised of diverse theories, methods, and practices (Wortley and Mazerolle, 2008; Schaefer et al., 2016), there is a central focus on the role of crime opportunities. One of the most notable illustrations of the importance of opportunity is observed in routine activity theory (Cohen and Felson, 1979). The theory outlines that crime occurs when three conditions converge in place and time: (1) a motivated offender (or an individual who becomes motivated when presented with an opportunity to offend), (2) a suitable target or victim, and (3) the lack of a capable guardian. Importantly, the everyday (generally non-criminal) behaviors of people bring them into contact with people, places, and precipitators that are criminogenic or help to protect them from exposure or vulnerability to such crime opportunities (Wortley, 2008). As such, individuals and organizations alike can develop very practicable solutions for disrupting these ingredients for crime, as the removal of even one element of this calculus is sufficient for prevention (Clarke and Eck, 2005). This simple but effective framework can have important implications for preventing reoffending among community corrections clients (Schaefer et al., 2016).

#### Environmental Corrections

Success may be limited when probation and parole authorities only generically address crime opportunities (Cullen et al., 2002; Schaefer et al., 2016), and evaluations show that vague deterrence tenets generally fail to reduce recidivism amongst community-supervised offenders (Cullen et al., 2017). Conversely, if community corrections agencies were to focus more seriously on the role of opportunity, the prospects for discouraging reoffending are made malleable. Merging the insights of environmental criminology with community corrections, the “Environmental Corrections” framework identifies two interrelated elements of opportunity-reduction that can improve probation and parole supervision. First, officers can engage offenders in interventions (through formal rehabilitative programming or through brief interventions conducted in routine case management meetings) that alter the ways they think about crime opportunities. Second, through targeted supervision conditions and revised routine activities, officers can restructure the kinds of opportunities for offending to which their supervisees are exposed. In this way, probation and parole officers act as problem-solvers who analyze each individual’s reoffending risks based upon (1) the kinds of opportunities that have proven tempting previously and (2) the opportunity structures with which they are in routine contact. Officers carefully examine each individual’s offense history (drawing on police accounts, court records, assessment results, and third party reports) to curate a risk profile of specific precipitators, and then imagine how these risks are embedded in space and time considering each client’s routine (as detailed in a weekly time diary, for instance; see Schaefer et al., 2016). With this information in hand, officers can then work to steer probationers and parolees away from situations that have been demonstrated to be criminogenic while providing them with new ways of thinking about the chances to reoffend that remain (Cullen et al., 2002).

#### Environmental Corrections and Other Evidence-Based Practices in Community Corrections

In the same way that we observe place-based crime concentrations, penologists have likewise observed that corrections outcomes (such as sentencing trends and re-entry and reoffending measures) demonstrate spatial aggregations (Rose and Clear, 1998; Clear et al., 2003; Clear, 2005; Sharlein and Engstrom, 2018), influenced by features of those communities (Baglivio et al., 2015; Chamberlain and Wallace, 2016). Given these associations, it is sensible that probation and parole officers occasionally utilize techniques that are opportunity-focused, such as through efforts to limit supervisees’ unstructured leisure time or associations with risky peers (Miller, 2014; Miller et al., 2015). The Environmental Corrections framework, first articulated by Cullen et al. (2002) and later elaborated by Schaefer et al. (2016), represents a shift from these *ad hoc* practices to a more formalized and systematic approach to the integration of opportunity-reduction into community supervision practices.

Likewise, there are clear examples of evidence-based practices in community corrections that share some of the elements utilized in Environmental Corrections, such as brief interventions focused on cognitive skills training [e.g., Staff Training Aimed at Reducing Rearrest (STARR; Robinson et al., 2011), Effective Practices in Community Supervision (EPICS; Smith et al., 2012), and Strategic Training Initiative in Community Supervision (STICS; Bonta et al., 2019)], and many departments integrate some degree of these models [e.g., the risk, need, and responsivity (RNR) principles (Andrews and Bonta, 2010), and core correctional practices (Dowden and Andrews, 2004)] into their routine practices. However, although the evidence-base of these effective practices has expanded substantially in the past few decades, leading penology scholars have argued that there remains a persistent “crisis in corrections” (Cullen and Jonson, 2016); agencies have been slow to incorporate organizing frameworks for their work, prioritize recidivism-reduction as the guiding philosophy, and use the best available evidence about the causes and prevention of crime (Cullen et al., 2017), at least in more methodological and systematic ways (Schaefer and Brewer, 2022).

Notwithstanding the contributions of other opportunity-reduction and targeted/tailored interventionist elements of the above-referenced innovations in probation and parole tactics, here we draw attention to the unique contributions of a model that more explicitly integrates the contributions of crime science into community corrections. Under an Environmental Corrections model, officers identify each probationer and parolee’s exposure and vulnerability to crime opportunities and then (1) develop supervision stipulations that limit that individual’s access to those opportunities, (2) create routines that implement these opportunity-reduction efforts while also placing the individual into patterned contact with prosocial people and activities (so as to encourage long-lasting behavioral change, whereby unwanted conduct is not just eliminated but replaced), and (3) work with the individual to build opportunity avoidance and opportunity resistance skills. Readers will note that traditional forms of probation and parole supervision likewise rely on supervision conditions to steer clients away from risks of reoffending. However, we contend that these case plan stipulations (behavioral restrictions and prescriptions) tend to be generic and oriented around loose deterrence tenets (e.g., supervisees must refrain from associating with other known offenders), while the Environmental Corrections model more narrowly identifies and stipulates conditions that are related to each individual’s risks for reoffending. Under an opportunity-reduction supervision framework, officers would tailor case plans to the unique opportunity-related risks that each client exhibits (e.g., creating rules around a specific co-offender or victim). In this way, generic conditions are replaced with specific conditions, which can be easier to define, monitor, and enforce (Cullen et al., 2002; Schaefer et al., 2016) and are thereby less restrictive than the broad stipulations ordinarily administered in probation and parole orders. An evaluation of a trial of Environmental Corrections has revealed that the opportunity-reduction framework can effectively reduce recidivism (Schaefer and Little, 2020).

### Current Study

In this study, we employed the quantitative and qualitative data from this same Environmental Corrections trial (Schaefer and Little, 2020) to focus explicitly on a subsample of probationers and parolees who are on community corrections orders for DFV offenses. Although there is ample evidence that opportunity-reduction tactics are reliably effective in crime prevention (Wortley and Mazerolle, 2008; Schaefer et al., 2016), and emerging evidence that these strategies may be useful for reducing reoffending in community corrections (Schaefer and Little, 2020), the reality is that DFV perpetrators present unique challenges for probation and parole authorities (Crowe et al., 2009; Spencer et al., 2020). Indeed, evidence demonstrates that many DFV perpetrators can at times engage in denial and victim-blaming behaviors (Henning et al., 2005), which can make preventing reoffending all the more difficult for officers required to discuss clients’ offending with them. This situation is complicated by the evidence that many of the formal treatment programs used by correctional authorities for their DFV clients are ineffective (and at times criminogenic; Welsh and Rocque, 2014; Graham-Kevan and Bates, 2020). Some scholars have even suggested that the political tensions involved in government and community responses to DFV discourage the use of evidence-based responses (Bates, 2016).

Drawing on these collective insights, we suspected that Environmental Corrections may have utility for the reduction of reoffending with DFV probationers and parolees because of its targeted focus on opportunity rather than propensity. The causes of DFV are complex and many contributing factors are outside the scope of what community corrections agencies can reasonably address in short, periodic supervision meetings (such as gender ideologies and cultural norms, structural sexism, and deep-seated perpetrator comorbidities; Graham-Kevan and Bates, 2020). Yet by providing probation and parole officers tools to control the other ingredient of crime events—opportunity—there are practicable ways we can prevent DFV perpetrators from reoffending. Moreover, because DFV perpetrators find *specific* crime opportunities tempting (i.e., a particular victim, or a reliable precipitator that triggers a cycle of violence), it is imperative that corrections authorities consider the unique risks of these clients and develop supervision strategies that are tailored to addressing those factors. In this way, we hypothesized that an opportunity-reduction supervision framework may hold promise for DFV offenders servicing corrections orders in the community.

Within the trial, officers sought to first understand the reoffending risks unique to each client, based on information drawn from their offense history. The officer would examine the available information (such as police reports and psychologist assessments) to determine the stimuli that triggered the decision-making to commit the offense. Officers then worked with the client to discern how they spent their time, envisioning how these crime precipitators could be routinely encountered or avoided in an average day or week. From there, the officer collaborated with the client to develop a schedule that would help them to steer clear of these known risks; sometimes these were suggestions (e.g., Joe is encouraged to spend time at his mother’s house or with his coach at the gym on Friday and Saturday evenings, which have historically led to idle time, “spiraling,” and poor decision-making), while in other instances enforceable supervision conditions were put into place (e.g., Jane is permitted to visit with her children only in the presence of Child Safety staff; Tom must not enter the 1,600 block of Smith Avenue and must stay 500 m from Sue). For each client, the goal was to develop *routine activities* that sidestepped known opportunity risks while also placing exposing them to prosocial influences. Within supervision meetings, officers then engaged clients in brief interventions oriented around cognitive skills trainings (e.g., self-control, consequential thinking, problem-solving) so that remaining risks could be resisted if encountered. Importantly, the case plan for each offender (inclusive of supervision conditions, in-house interventions, and outsourced services and programs) was based on their individual risks for reoffending, although officers were encouraged to consider the available evidence about the known risk, criminogenic need, and specific responsivity considerations relevant to particular offense types (such as DFV).

## Methodology

The Environmental Corrections framework was implemented in one Community Corrections office in a large metropolitan Australian city for 6 months in 2016. Our data for the current evaluation were drawn from this trial, where all staff and all offenders at the experimental site participated. Staff received a 2-day training on the model, including a coverage of opportunity-reduction theories and their relevance to probation and parole, tools for assessing opportunity-related risks and developing supervision conditions and new routines, and rehearsal of the techniques used in brief interventions that use cognitive skills training (oriented around opportunity identification, avoidance, and resistance). Consistent with environmental criminological theories and tactics, officers learned how to identify and interrupt these convergences of prospective offenders and suitable opportunities (examples of some of the tools used in the trial can be found in Schaefer et al., 2016). A researcher on the project attended the trial office to make observations and answer questions, performing booster trainings roughly monthly to aid in model fidelity.

To make inferences about the effects of the experimental intervention on reoffending, a comparison site (a Community Corrections office in geographic proximity with comparable client and offense characteristics) was selected to serve as the control group (see Table 1; for a fuller description of the participants at both sites, see Schaefer and Little, 2020). The comparison site continued to deliver probation and parole supervision as “business as usual,” utilizing a framework best characterized as a compliance-oriented and administratively focused form of case management. These routine practices did not contain any substantive elements of opportunity-reduction supervision or some of the leading evidence-based programs in community corrections (such as EPICS, STARR, or STICS).

**TABLE 1:** Comparison of Treatment and Control Groups.

	Whole treatment group		Whole control group		Hypothesis test		DFV treatment group		DFV control group		Hypothesis test	
	(*n* = 1,681)		(*n* = 1,296)				(*n* = 194)		(*n* = 187)			
						
	P/M	*SD*	P/M	*SD*	Test	Sig.	P/M	*SD*	P/M	*SD*	Test	sig.
Individual characteristics												
Age	31.96	10.25	32.61	9.98	*t* = -1.738	0.082	33.55	9.70	33.57	8.50	*t* = -0.022	0.983
Sex (0 = male)					χ^2^ = 0.908	0.341					χ^2^ = 7.286	0.007
Male	0.78		0.77				0.91		0.82			
Female	0.22		0.23				0.89		0.18			
Indigenous status					χ^2^ = 44.720	0.000					χ^2^ = 0.437	0.508
Aboriginal or Torres Strait Islander	0.77		0.87				0.18		0.16			
Not Aboriginal or Torres Strait Islander	0.23		0.13				0.82		0.84			
Risk characteristics												
Risk of reoffending score	10.22	8.93	9.73	5.53	*t* = 1.740	0.082	11.82	3.93	10.57	3.96	*t* = 3.053	0.002
Level of service					χ^2^ = 17.694	0.001					χ^2^ = = 4.998	0.082
Low	0.09		0.11									
Standard	0.38		0.44				0.43		0.55			
Enhanced	0.34		0.30				0.37		0.30			
Intensive	0.19		0.15				0.20		0.15			
Correctional characteristics												
Length of order (in months)	19.43	60.38	20.00	78.22	*t* = -0.224	0.823	10.46	6.32	11.72	6.22	*t* = -1.944	0.053
Number of prior orders	1.96	2.74	1.64	2.24	*t* = 3.463	0.001	2.20	3.48	1.37	1.86	*t* = 2.833	0.005

### Participants

Across the entire duration of the trial, the treatment group included 1,681 offenders, and the control group was composed of 1,296 offenders, for a total sample size of 2,977. For the majority of the quantitative analyses in this study, we were interested primarily in those individuals serving probation and parole orders who had DFV listed as their *index offense*. Individuals who were serving orders for other offenses that may have included DFV (e.g., stalking, assault, homicide, incest) were not included in these categorizations due to limitations in the data provided by Corrective Services for our evaluation. To help address this shortcoming, for the qualitative analyses in this study, we analyzed all interview transcripts of participants in the sample who *self-disclosed* DFV as being a *component* of the offenses that led to their current order, even in the event that DFV was not their index offense.

Including only those orders for DFV index offenses during the trial window, the total treatment group included 194 offenders (11.54% of the Office cohort) and the total control group included 187 offenders (14.43% of the Office cohort), for a total subsample size of 381 (noting that in any given month, fewer individuals may be on orders at that time due to differing times of order commencement and completion). Of these individuals, the overwhelming majority were male (86.61%) and not Indigenous (83.20%). At the completion of the trial, the average age of the subsample was 33.56 (*SD* = 9.12). Most of the probationers and parolees had served orders previously (60.89%; *M* = 2.16 previous orders, *SD* = 1.69). The average sentence length for the orders was roughly 1 year (*M* = 359.66 days, *SD* = 192.85). Using the agency’s standardized measure for assessing risk of reoffending (an actuarial tool assessed as a valid and reliable indicator of recidivism risk), the mean risk score was 11.16 (*R* = 1–20 (20 being the highest level of risk), *SD* = 4.00). These risk scores indicate the client’s level of service, which is then related to supervision intensity (such as reporting frequency). About half of the subsample was on standard level of service (48.03%; generally equates to monthly reporting), with smaller proportions on enhanced (33.07%; fortnightly reporting) and intensive (17.32%; weekly reporting) levels of service.

A comparison of the treatment and control group participants across these characteristics is showcased in Table 1. Comparing the entire treatment site (*n* = 1,681) vs. the control site (*n* = 1,296), the control site had a larger proportion of Aboriginal and Torres Strait Islander clients (87% vs. 77%; *x*^2^ = 44.720, *p* < 0.001) and clients on average had fewer prior orders (*M* = 1.96, *SD* = 2.74 vs. *M* = 1.64, *SD* = 2.24; *t* = 3.463, *p* < 0.001); across the two sites, there was also significant variation in the clients’ level of service (*x*^2^ = 17.694, *p* < 0.001). Looking at only DFV clients, in relation to the comparison site (*n* = 187), the treatment site (*n* = 194) included a higher proportion of male probationers and parolees (91% vs. 82%; *x*^2^ = 7.286, *p* < 0.01), a higher risk of reoffending score (*M* = 11.82, *SD* = 3.93 vs. *M* = 10.57, *SD* = 3.96; *t* = 3.053, *p* < 0.01), and more previous orders (*M* = 2.20, *SD* = 3.48 vs. *M* = 1.37, *SD* = 1.86; *t* = 2.833, *p* < 0.01). There were no statistically significant differences between the two DFV client groups with respect to age, Indigenous status, level of service, or sentence length. We return to these differences in our limitations subsection.

### Data Collection and Analytic Procedures

Our quantitative analyses were performed using official data from the Department of Corrective Services. Unfortunately, the data available for analysis extends only to the completion of the trial (the 6-month intervention window), so we are unable to calculate the effect of the intervention across a longer follow-up period. However, this still provides a reasonable snapshot of the immediate effects of the intervention on offending behaviors. Our primary dependent variable of interest for this study is reoffending, which was operationalized as a new offense as recorded by police. Although this measure provides a good indication of reoffending, it is important to note that (1) it does not capture criminal activity undetected by police and (2) it may include individuals who have the charges dropped or are found not guilty. Experts acknowledge that the actual prevalence of DFV is unknown (Mouzos and Makkai, 2004), although rates of DFV offending are likely to be much higher than what is known to police (Australian Institute of Health and Welfare, 2019). Readers should also note that our reoffending measure relates to *any* criminal charge. The data made available to the research team did not enable us to explore whether the reoffence related to further DFV behavior.

The qualitative analyses in this study were conducted with transcripts from semi-structured interviews performed with participants in the Environmental Corrections trial. Approximately 3 months post-intervention, data collection took place across a 2-week window. During this period, as required by Corrective Services, officers extended an invitation to participate in the interview to the probationer or parolee following their routine case management meeting. When individuals expressed interest in participating in the study, a trained Research Assistant met with the individual in a private room, provided information about the project and obtained informed consent, then completed the interview and debriefing procedures. In total, 119 invitations were issued and 53 were accepted, for a response rate of 44.54%. Of these 53 interviews, for this study we isolated a subsample of the participants who identified DFV as a component of the offending episode that led to their current probation and parole order (*n* = 10; 18.87% of the interview participants). This included 3 participants who had DFV as their index offense, three participants who had a breach of their DVO as their index offense, and 4 respondents who self-reported DFV as part of the reason for their current order. Brief participant characteristics are listed in Table 2.

**TABLE 2:** Interview participant characteristics.

Participants pseudonym	Sex, age	Order type	Order length	Index offence	Reporting frequency
Bill	Male, 25	Probation	18 months	Driving under the influence	Fortnightly
Dave	Male, 27	Probation	18 months	Breach of domestic violence order	Fortnightly
Frank	Male, 38	Probation	18 months	Domestic violence	Weekly
Holly	Female, 31	Probation	18 months	Assault	Weekly
Jake	Male, 31	Probation	12 months	Breach of domestic violence order	Monthly
Luke	Male, 35	Board-ordered parole	9 months	Domestic violence	Weekly
Nate	Male, 37	Intensive corrections order	6 months	Domestic violence	Weekly
Pete	Male, 47	Probation	6 months	Breach of domestic violence order	Monthly
Rick	Male, 48	Probation	9 months	Alcohol-related violence	Monthly
Tony	Male, 53	Court-ordered parole	3 months	Driving under the influence	Fortnightly

The interviews were semi-structured, following an interview guide with questions and prompts related to the participant’s history of offending, experiences on probation or parole, efforts to desist, and goals/expectations for the future. The interviews ranged from 12 to 48 min (*M* = 23, *SD* = 10) and were audio recorded, transcribed verbatim, and de-identified for the analyses used in this study. The interview transcripts were analyzed according to Braun and Clarke’s (2006) six-step method for thematic analysis, whereby we (1) became familiar with the data, (2) developed initial codes, (3) searched for themes, (4) reviewed the themes against the data and revised them as needed, (5) named and defined the themes, and (6) wrote our results. Given our understanding of the Environmental Corrections trial in which these interviews were situated, for these analyses, we extracted interview excerpts that related to crime opportunities generally and sought to contextualize these findings in the broader literature of environmental criminology and opportunity reduction. From these excerpts, our analytic approach embraced a phenomenological, grounded framework whereby themes emerged organically from the data through the iterative process described above.

## Results

Domestic and family violence (DFV) perpetrators present unique challenges for probation and parole authorities who supervise these individuals in the community (Crowe et al., 2009; Spencer et al., 2020). In this study, we speculated that the Environmental Corrections framework (Schaefer et al., 2016) may be useful with this cohort of offenders compared to other offender types in the same jurisdiction, given its emphasis on targeted opportunity-reduction strategies rather than generic deterrence tactics or exclusive efforts to address criminal propensity (especially given that DFV clients are likely to have complex criminogenic needs; Crowe et al., 2009; Graham-Kevan and Bates, 2020; Spencer et al., 2020). A previous evaluation of a trial of Environmental Corrections demonstrated a 28% recidivism reduction for the treatment group compared to a propensity score matched control group (Schaefer and Little, 2020) and revealed several important benefits for the clients supervised under this model (Williams and Schaefer, 2021). Here, we analyze these same data (although we were unable to use comparable propensity score matching techniques) using a mixed methodology to explore whether the model has any unique relevance for DFV probationers and parolees.

### Quantitative Analyses

Across *all* probationers and parolees, following 6 months of the Environmental Corrections intervention, 32.39% of the control group participants incurred a police-recorded reoffence (for any crime type, not just DFV), compared to 26.50% of the treatment group. This represents a 18.18% raw recidivism reduction, which was statistically significant at the *p* < 0.05 level (χ^2^ = 4.905). Performing the same calculations with only those individuals serving probation and parole orders for DFV index offenses, at the completion of the trial, 32.84% of the control group had reoffended, compared to 27.78% of the participants in the experimental condition. Although DFV probationers and parolees had slightly higher reoffending rates than other offenders (i.e., those on community supervision orders for other offense types) at both the treatment site (χ^2^ = 0.061, *p* = 0.804) and the comparison site (χ^2^ = 0.010, *p* = 0.921), these were not meaningful differences. Looking at only those clients on orders for DFV offenses, there was no significant difference in rates of contravention (technical violations of supervision conditions that do not constitute a new offense) between the control group (17.70%) and the treatment group (15.56%; χ^2^ = 0.970, *p* = 0.325).

**FIGURE 1:**
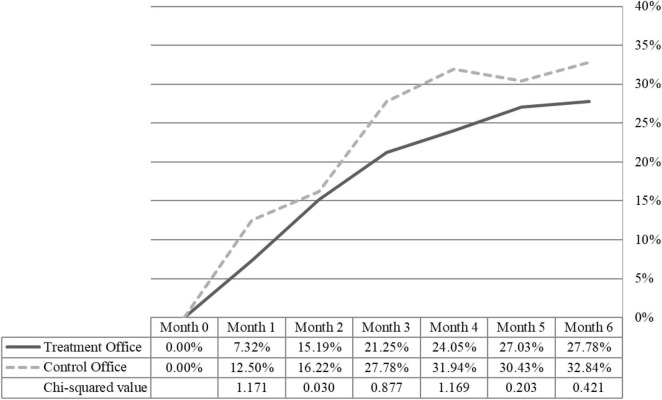
Cumulative rates of reoffending post-intervention.

The cumulative rates of reoffending amongst DFV offenders plotted in Figure 1 demonstrate a 15.41% reduction in recidivism between the two groups, although the difference was not statistically significant (χ^2^ = 0.421, *p* = 0.516). We speculated that these raw differences in reoffending across the two groups at the completion of the trial perhaps failed to reach statistical significance given the smaller subsample for the DFV probationers and parolees compared to all offenders. We further considered that there may be unaccounted for differences between the two groups (given that this was a quasi-experiment with no random sampling or assignment; see Table 1). We therefore performed a binary logistic regression to determine whether these small group differences would reach significance after statistically controlling for several important covariates of risk. As seen in Table 3, although the odds of a police-recorded reoffence (for any criminal charge) at 6 months post-intervention were 21.50% lower for DFV Environmental Corrections trial participants, this was not a statistically significant difference even in light of the other variables in our model. The only significant predictor was sentence length, with the odds of reoffending increasing for longer orders, perhaps indicative of the effects of exposure or “street time” (given that the risk of reoffending score was not statistically significant).

**TABLE 3:** Binary logistic regression predicting reoffending.

	exp(β) (S.E.)	Sig.
Individual characteristics		
Age	0.949 (0.031)	
Sex (0 = male)	1.248 (0.594)	
Indigenous status (0 = not Aboriginal or Torres Strait Islander)	1.167 (0.507)	
Risk characteristics		
Risk of reoffending score	1.015 (0.059)	
Number of risk domains assessed as high-risk	0.888 (0.098)	
Correctional characteristics		
Length of order	1.002 (0.115)	*
Number of prior orders	1.004 (0.115)	
Environmental corrections participant (0 = no)	0.785 (0.394)	
Model information		
Intercept	0.893 (1.459)	
-2 log likelihood	160.061	
Model χ^2^	17.762	*
Nagelkerke *R*^2^	0.101	
*N*	381	

*p < 0.05.

### Qualitative Analyses

We performed a thematic analysis of 10 interview transcripts from probationers and parolees in the Environmental Corrections trial who had DFV involvement in their current offense/order. We extracted all interview excerpts that related to the role of opportunity (*n* = 148) and categorized these into three themes: causes of offending (*n* = 31; 20.95%), desistance from offending (*n* = 65; 43.92%), and supervision (*n* = 52; 35.14%). From there, we further coded each excerpt into relevant subthemes. In the subsections that follow, we unpack some of these subthemes (noting that they may tally to greater than 100%, as an excerpt within a theme may be related to more than one subtheme), using pseudonyms to identify participants.

#### Causes of Offending

Of the 31 interview excerpts related to our participants’ (perceived) causes for their DFV offending, four subthemes emerged: routines, anger, reversal, and reframing. Most notably, respondents identified *routines* (*n* = 12; 38.7%) as being more or less criminogenic. This may have been due to boredom (“Now I’m working and stuff, I’m not at home all the time now and just constantly thinking about stuff. I’m not bored anymore, so I think most of it [offending] was because of boredom.”—Dave), such that keeping busy is associated with breaks in offending (“Boredom, to escape reality…[now] I start thinking about doing something around the house, fixing something, going to the park, kicking the footy around.”—Frank). However, many identified that *anger* (*n* = 8; 25.8%) could override these intentions (“When you’re mad you do things that you wouldn’t normally do.”—Bill), particularly in ways that seemed to short-circuit their ability to exercise foresight (“I get really angry and I don’t really think about the long run. I don’t really think of what’s going to happen after the—I don’t think of the consequences or anything. I just go and do it. Then once I’ve done it and I’ve been in trouble, I think, ‘Oh my god’.”—Holly).

Although these two subthemes revealed important insights into the catalysts of crime, our participants also showcased forms of denial about their offending, such as through *reversal* (*n* = 7; 22.6%), in which the perpetrator blamed the victim for the offense (“Well most of it was false accusations…because she was envious of me and [my relationship with] the kids.”—Frank) and saw themselves as the aggrieved (“I know everyone says they’re innocent, but what happened to me wasn’t my fault. I mean, this person - I didn’t want to see him anymore. He came in and broke into my house.”—Tony). On a few occasions, individuals did not turn the tables as such, but engaged in a *reframing* (*n* = 5; 16.1%) of their offense. This came through when individuals tried to distance their current self from past indiscretions (“Look, I did [assault her] ages ago when we first started going out. I did in the first couple of years but not recently like she was stating.”—Rick), or through impression management efforts to justify their behavior (“I was just mostly mad at myself, because I wasn’t supporting my girlfriend and bringing in any income or anything to the house. So that’s what also made me mad, but it wasn’t at her. It was just at myself and I’d snap but it wasn’t meant to be a snap towards anybody.”—Dave). Combined, these excerpts showcase that the probationers and parolees interviewed as part of the trial may have some important understandings about the causes of their DFV, but that inappropriate and inaccurate rationales remained. Critically, however, this may not be an issue insofar as the Environmental ‘Corrections framework emphasizes opportunity-reduction rather than propensity-reduction, a point we return to in our discussion of these results.

#### Desistance From Offending

Our analyses revealed 65 interview excerpts that described participants’ efforts to desist from offending, again with four subthemes observed in the data: social supports, routine, avoidance, and consequential thinking. Most commonly, respondents highlighted the role of *social supports* (*n* = 27; 41.5%) in motivating their efforts to cease undesirable behaviors (“It’s mainly because of my kids.”—Luke). Their family and friends facilitated desistance in multiple ways, ranging from emotional support (“Some do it by not acknowledging it and ignoring it and just treating me like a normal person. Some do it by getting up you all the time. Some do it by being compassionate. They all do it differently.”—Jake), to concrete support (“They’ve given me a place to go where I can get food and stuff…Showing me where to go to get Medicare cards and how to go about going to a GP and stuff like that. Because there’s a lot of this stuff I don’t know.”—Bill), to loved ones acting as “crime controllers” (“My mate’s good for it, yeah. I’ll just sit there and drink mine slower and once he knows I’ve had a couple, once he sees I’ve had two he says, ‘Oh let’s go, we’ll go now’.”—Pete). Our interview participants likewise highlighted how a new *routine* (*n* = 24; 36.9%) had helped them to reduce their reoffending, through general busyness with prosocial goals (“My lifestyle is quite busy now. I keep myself occupied with good things. I have goals now, whereas before, I didn’t.”—Tony) and through (often new) prosocial peer influences (“I’m in a happy space because a lot of people around me are in a happy space.”—Rick).

More narrowly, some individuals identified how they have varied their routines to use *avoidance* to their benefit (*n* = 19; 29.2%), which at times overlapped with routines (“Instead of going to the pub every afternoon, I take my dog for a work or I’m out in the garden or whatever.”—Tony) and certain criminogenic associates or environments (“I don’t hang out in those places where they are, houses, clubs or where they do [drugs] - so, I pretty much don’t see them, all those people anymore.”—Rick). Some of our respondents highlighted how the *consequential thinking* (*n* = 4; 6.2%) skills they learned during the Environmental Corrections trial contributed to the improvements they observed (“Look at the options, look at the reactions to which one you choose, and choose the best one.”—Jake). Across these subthemes, our participants were able to identify and articulate various motivations and mechanisms for preventing their own offending which often showed areas of overlap with the evidence of how to prevent crime through the reduction of opportunities.

#### Supervision

We extracted 52 interview excerpts from our DFV participants that related to the theme of supervision. Of those, two subthemes emerged: intervention and routine. First, our respondents frequently discussed the role of *intervention* (*n* = 40; 76.9%) in their case management meetings and the effects these mini-treatment efforts were having on their day-to-day life. Many individuals described particular cognitive skills trainings they did with their supervising officer that related to anger and/or consequential thinking (“She gets me to do this five-step thing when I usually get angry, because I usually used to get angry and just snap for no reason…[Previously] I just went off and that was it. That’s pretty much why I ended up here really.”—Dave). Some of our respondents related these interventions to their DFV perpetration specifically. For instance, Holly described:

“We put a goal plan in place. Talked about our goals and to try and think ahead. We wrote a goal sheet thing out. Really big piece of paper. She just put lots of goals and then facts. For me to think ahead of my actions instead of actually following through with them. Because when I get really angry and in that moment I get, I aim to - I could do anything silly and it will just breach my DVO. It would breach my thing. So she tries to teach - tell me to think before I actually take action. Just think of the pros and cons. If I’m going to go over there just try and think about what’s going to happen if I did go over there.”

Participants also noted how their case management meetings were used to discuss and modify their *routine* (*n* = 13; 25.0%), which helped them to identify and navigate some of the lifestyle factors that may have led to their offending in the first place (“When I first came out, I’d run into certain people and they would talk with me… it’s easy to fall because that’s when I felt most risk…but I got a good corrections officer - otherwise I could’ve just gone and just done my thing again.”—Rick).

Taken together, these two subthemes of routine and intervention in relation to supervision highlight the impact of opportunity-reduction supervision for these probationers and parolees. For instance, although *routine* was a subtheme in the interview excerpts related to the causes of offending, it also emerged as a feature of supervision; indeed, some observed how the lack of a structured schedule may be criminogenic but that their officer was assisting with that (“A lot of people that do offences don’t have any sort of routine, so it’s [supervision requirements] the first step of having a routine.”—Jake). Although the probationers and parolees supervised at the Environmental Corrections site were not made aware of the experimental trial, some of the interview participants were able to identify a clear shift in supervision practices based on the more interventionist style of case management they were receiving from their officer. For example, participants described how, “[Officer] asked me have I had any problems in the last 2 weeks where I thought I could’ve made a better decision type thing. I’ve never experienced that before from a parole officer.” (Tony), “Just how now we sort of write things down and go through them and set goals to aim for. Whereas before it was just like she just keeps asking how things are and what I’ve been up to rather than pinpointing certain issues and things that are going on.” (Luke), and “Before you just go in and sign your paper or whatever and stand for 5 minutes and just walk off. Now, they actually like – It’s good. They do activities for you and stuff to get into if you actually want to help yourself.” (Nate). It seemed evident in our data that the clients involved in the trial were able to identify a difference, and that many of them felt these changes were beneficial.

## Discussion

Corrections departments are managing record numbers of offenders, driven in part by high rates of recidivism (Australian Government Productivity Commission, 2021). At the same time, government agencies are facing increased political and public pressure to address DFV. At the crossroads of these realities are community corrections officials who must navigate the supervision of DFV probationers and parolees, many of whom exhibit unique risks and challenging criminogenic needs (Crowe et al., 2009; Graham-Kevan and Bates, 2020; Spencer et al., 2020). These individuals may not be randomly motivated when crime opportunities present themselves but may instead be more predatory; there are *specific* precipitators that community corrections officers must be mindful of, so generic deterrence strategies may not be effective with this cohort. Studies show that formal interventions with DFV perpetrators are often unsuccessful (Graham-Kevan and Bates, 2020), in part because they may be inclined to minimize or deny their misconduct or blame the victim (Henning et al., 2005), making it difficult for officers to have meaningful conversations with offenders about their behavior and efforts at reform.

For these reasons, we speculated whether a different tactic may be useful. Rather than officers focusing too heavily on propensity (which is deep-rooted and can be difficult to change in the context of brief, periodic supervision meetings)^4^ or deterrence (in the form of loose supervision conditions accompanied by the threat of subsequent punishments), perhaps greater success could be observed if we focus on the external situations that convert or catalyze underlying motivation into criminal activity. Recent advances in community corrections scholarship show that opportunity-reduction tactics may be useful in steering offenders away from chances to reoffend (Miller, 2014; Miller et al., 2015; Schaefer and Little, 2020), an important divergence from mainstream supervision frameworks that rely on generic deterrence strategies or bureaucratic forms of case management that emphasize order compliance rather than behavioral change (Cullen, 2002; Bonta et al., 2008; Pew Center on the States, 2008; Taxman, 2011; Burrell, 2012; Schaefer and Brewer, 2022). Merging the theories and methods of environmental criminology with community corrections, Schaefer et al. (2016) have proposed an “Environmental Corrections” framework. Under this model, officers create new routine activities for offenders that keep them away from unique risks to reoffend while also increasing their exposure to prosocial influences. At the same time, officers intervene with their supervisees to develop cognitive behavioral skills in identifying, avoiding, and resisting the crime opportunities that are bound to remain in each client’s environment. In this study, we utilized a mixed-methodology to evaluate the utility of this model with DFV probationers and parolees.

Using a quasi-experimental design, our quantitative analyses revealed that the Environmental Corrections trial reduced police-recorded reoffences by 18.18% in the treatment group compared to the control group when examining *all* offenders. Examining only DFV offenders, the difference in the rate of reoffending at 6 months post-intervention reduced to 15.41% (whereby 27.78% of the treatment group reoffending compared to 32.84% of the control group). We believe that this reduction is substantively meaningful although not statistically significant (for discussions of the interpretation and limitations of *p*-values, see Amrhein et al., 2019; Kuffner and Walker, 2019), perhaps highlighting the need for additional trials and evaluations with larger sample sizes and an extended follow-up window. It is important to point out that the rates of reoffending did not differ significantly between DFV offenders and all other offenders at the treatment or comparison site, and that there were no significant differences in rates of technical violations. As such, although our findings do not meet the traditional thresholds for statistical significance used in the social sciences, the results do indicate that opportunity-reduction supervision may hold promise in minimizing reoffending among DFV probationers and parolees. Accordingly, we contend that these results are of substantive significance, especially considering the impacts and harms resulting from DFV (Rollé et al., 2019).

Given these results, we examined the perspectives of DFV probationers and parolees at the trial site to explore the impact of the Environmental Corrections framework. We analyzed the transcripts from semi-structured interviews with 10 supervisees who identified DFV as a component of their offending that led to their current order. Our participants highlighted many of the evidence-based theories and practices used in environmental criminology and crime science, such as the role of routine activities in being more or less criminogenic, the utility of avoidance (of risky people, places, and provocations), and the impact of “offender handlers” (Clarke and Eck, 2005; Wortley and Mazerolle, 2008; Clarke, 2010; Sampson et al., 2010; Schaefer et al., 2019). The brief interventions performed in officer-offender meetings also appeared to be influential, with several of our participants articulating the everyday uses they were finding for these cognitive skills (such as consequential thinking).

Research demonstrates that many DFV perpetrators at times exhibit minimization, denial, and victim-blaming (Henning et al., 2005), and many of our formal rehabilitation efforts with these correctional clients are ineffective (Graham-Kevan and Bates, 2020). Indeed, our thematic analysis revealed that some of the DFV probationers and parolees used reversal and reframing to sidestep the root causes of their misconduct, although others indicated that anger and poor consequential thinking are related to their offending. Partly for this reason, we speculated that an opportunity-reduction supervision framework may be useful with DFV clients (compared to other offense types in the same jurisdiction), given its focus on opportunity rather than propensity. We speculated that targeted supervision conditions could help to keep DFV offenders away from real risks for reoffending, rather than trying to alter their criminal etiology or convince/threaten them to make more rational (prosocial) choices. For instance, an evaluation of the impact of community treatment on male batterers showed that they were not deterred by expectations of formal or informal consequences, and that motivation was unrelated to recidivism (Hanson and Wallace-Capretta, 2004). Although common “treatment speak” endorses the notion that individuals must recognize their problematic behavior and its roots in order to effectively address it, this is at odds with the evidence about the efficacy of opportunity-reduction crime prevention (and the limited function of motivation) more broadly.

All of these findings combined, we suggest that opportunity-reduction frameworks may be useful for the community supervision of DFV probationers and parolees, although further evaluation is needed. We are inclined to believe that dosage may be important here, although more extensive trials would be required to confirm. Given that the trial was only 6 months long, offenders may have had minimal contact with their supervising officer (e.g., a DFV probationer on monthly reporting would have had only six case management meetings). Our data revealed that more visits to the probation and parole office during the trial was associated with *more* reoffending (*t* = -5.260, *p* < 0.001), although this is likely due to the confounding effects of reoffending risk levels. We have reason to believe that an opportunity-reduction framework for community supervision may be more or less effective for certain types of offenses and/or correctional clients, although the data for the current study do not allow us to sufficiently attend to some of these hypotheses. A rigorous experimental trial would help to resolve some of the questions that our data do not enable us to answer. In any event, the growth curves in Figure 1 appear to demonstrate that opportunity-reduction supervision tactics limit reoffending compared to business-as-usual in probation and parole.

### Limitations and Directions for Future Research

The contributions of our study notwithstanding, there are several shortcomings that contextualize our results, providing important implications for further study. As described above, we had limitations in our measurement window, sample sizes, and ability to fairly compare the treatment group and the comparison group. Indeed, some of the differences between the two groups may have been due to underlying characteristics rather than the effects of the pilot test (see Table 1), and it may be that “doing something” was better than “doing nothing” (i.e., business-as-usual) rather than specific elements of Environmental Corrections. An experimental trial (or at a minimum, a more rigorous quasi-experiment) would help to address these complicating factors. Our quantitative analyses would have benefited from a longer follow-up period and more fine-grained measurements of DFV. Additionally, since the prevalence of DFV is difficult to estimate (Mouzos and Makkai, 2004; Australian Institute of Health and Welfare, 2019), additional measures of reoffending (such as self-report data in addition to reconviction and reimprisonment variables) are required. Although the sample size for our qualitative analyses is acceptable for studies that explore the commonalities of lived experience in a somewhat homogenous sample (Creswell, 1998; Kuzel, 1999), additional qualitative investigation is necessary. Specifically, we urge researchers to sidestep the inclination to exclusively investigate the motivations for DFV perpetration, and more thoroughly explore the role of crime opportunities in facilitating and preventing these (re)offenses.

### Implications

Although further research is required, the results of our study provide preliminary and cautious support for the notion that the supervision of DFV probationers and parolees could be augmented by moving beyond a compliance-oriented model (Spencer et al., 2020). According to the principles of effective correctional intervention (Andrews and Bonta, 2010), the risk principle stipulates that the intensity of the intervention should be commensurate with the offender’s degree of risk (which should be gauged through actuarial assessment). Although this principle is often interpreted in the context of treatment (i.e., high-risk offenders require more intensive treatment than low-risk offenders), it can also be applied through the lens of supervision intensity (Schaefer and Brewer, 2022). However, rather than having control-focused conversations, officers can make meaningful impacts with their supervisees when the discussion is centered around behavior change (Bonta et al., 2008; Taxman, 2008; Robinson et al., 2012; Smith et al., 2012). We believe that the findings of our study demonstrate the potential value of altering unique risk-related behaviors for individual DFV offenders through a framework of opportunity-reduction supervision. Rather than efforts that exclusively seek to alter the motivations or etiology of DFV probationers and parolees or emphasize generic deterrence tenets, research should further investigate the utility of officers working with these offenders to minimize opportunities for reoffending in a targeted and tailored way.

## Data Availability Statement

The datasets presented in this article are not readily available because data are available only to authorized users as granted by the Department of Corrective Services. Requests to access the datasets should be directed to corresponding author.

## Ethics Statement

The studies involving human participants were reviewed and approved by the Griffith University Human Research Ethics Committee. The patients/participants provided their written informed consent to participate in this study.

## Author Contributions

LS, GW, and EM contributed to conception and design of the study. LS performed the statistical analyses. GW developed the qualitative methodologies used in the project. LS wrote the first draft of the manuscript with subsections authored by EM and GW. All authors contributed to manuscript revisions, and have read and approved the submitted version.

## Conflict of Interest

The authors declare that the research was conducted in the absence of any commercial or financial relationships that could be construed as a potential conflict of interest.

## Publisher’s Note

All claims expressed in this article are solely those of the authors and do not necessarily represent those of their affiliated organizations, or those of the publisher, the editors and the reviewers. Any product that may be evaluated in this article, or claim that may be made by its manufacturer, is not guaranteed or endorsed by the publisher.

## Footnotes

1
https://www.march4justice.org.au/

2 Unfortunately, these figures do not consider the population size of the state. Estimates by the Australian Bureau of Statistics indicate that the population of Queensland was 3.67 million in 2001 compared to 5.22 million in 2021, suggesting that the underlying *rate* of breaches has grown in ways that outpace population growth.

3 While the current *National Plan* is set to expire in mid-2022, a renewed policy framework (for 2022–2032) has been drafted.

4 We are not discounting the importance and potential utility of formal rehabilitation efforts, such as standardized and validated curriculum-based programs or treatment efforts performed by qualified personnel (e.g., forensic psychologists, psychiatrists, substance abuse counselors). Rather, here we wish to emphasize the role of probation and parole staff in routine supervision meetings with their clients.
